# Microalgal lipid production using the hydrolysates of rice straw pretreated with gamma irradiation and alkali solution

**DOI:** 10.1186/s13068-015-0308-x

**Published:** 2015-08-27

**Authors:** Min-Ho Joe, Ji-Youn Kim, Sangyong Lim, Dong-Ho Kim, Suk Bai, Hyun Park, Sung Gu Lee, Se Jong Han, Jong-il Choi

**Affiliations:** Department of Biotechnology, Korea Atomic Energy Research Institute, Jeongeup, 580-185 Republic of Korea; School of Biological Sciences and Biotechnology, Chonnam National University, Gwangju, 500-757 Republic of Korea; Department of Biological Sciences, College of Natural Sciences, Chonnam National University, Gwangju, 500-757, Republic of Korea; Korea Polar Research Institute, Incheon, 406-840 Republic of Korea; Department of Biotechnology and Bioengineering, Chonnam National University, Gwangju, 500-757 Republic of Korea

**Keywords:** Rice straw, Pretreatment, Microalgae, Gamma ray irradiation, Lipid

## Abstract

**Background:**

Lignocellulosic biomass has long been recognized as a potential sustainable source of sugars for biofuels. However, many physicochemical structural and compositional factors inhibit the enzymatic digestibility of the lignocellulosic biomass. In this study, efficient pretreatment method of rice straw (RS) was developed and the RS hydrolysate was applied in the cultivation of microalgae for lipid production.

**Results:**

Gamma ray irradiation (GRI) and alkali solution were used for the pretreatment, and saccharification was carried out with lignocellulolytic enzymes. When RS was pretreated by combined GRI and alkali method, the glucose and xylose saccharification yield after enzymatic hydrolysis increased up to 91.65 and 98.84 %, respectively. The enzymatic hydrolysate from the RS pretreated with the combined method was used to cultivate *Chlorella protothecoides* for lipid production. The maximum concentrations of biomass and fatty acid methyl ester of cells were 6.51 and 2.95 g/L, respectively. The lipid content of *C. protothecoides* from RS hydrolysate was comparable to that from glucose, and the lipid composition was similar between different carbon sources.

**Conclusion:**

These results demonstrate that the combined pretreatment with gamma irradiation was highly effective in preparing hydrolysate, and the rice straw hydrolysate could be used as an alternative carbon source for microalgal lipid production for biofuel.

## Background

Lignocellulosic biomass, which is composed of cellulose, hemicellulose and lignin, has long been recognized as a potential sustainable source of sugars for biofuels and value-added biomaterials [1, 2]. However, many physicochemical structural and compositional factors inhibit the enzymatic digestibility of the lignocellulosic biomass that liberates the sugars necessary for fermentation [2]. Therefore, pretreatment is an essential processing step to break the lignin and to expose cellulose and hemicelluloses for enzymatic digestion. Among the various types of lignocellulosic biomass, rice straw (RS) is a potent candidate for biofuel production since it is one of the most abundant renewable resources in the world [3–5]. However, there is very little information available regarding the biodiesel production using this lignocellulosic biomass. In addition, there are different types and varying amounts of other compounds besides sugars, such as acetic acid, furfural, hydroxymethylfurfural, heavy metals and water-soluble lignin, which are known to inhibit microbial metabolism [6]. But, the content of these inhibitors and the effect of pretreatment on these are not well investigated.

Various pretreatment methods, which include physical, chemical and biological pretreatments, have been developed to enhance the efficiency of enzymatic hydrolysis and to lower the cost of biofuel production using lignocellulosic biomass [4, 5]. Although there are a number of reports on various pretreatment options, none of them can be considered as the ideal option because the choice of optimal pretreatment method depends on the type of biomass, and its economic and environmental impact [7]. Ionizing radiation, such as gamma rays and electron beam, can modify and disrupt the structure of lignocellulose by penetrating photons into the lignocellulosic structure and producing free radicals. Irradiation reduced the crystallinity of lignocellulose biomass, leading to a decrease in the degree of polymerization and increase in the surface area, which in turn increase the enzyme accessibility and enzymatic digestibility. For this reason, irradiation pretreatment has been investigated for decades as a physical pretreatment method of biomass [8–13]. Even though irradiation could be simply applied to all type of lignocellulose biomass with large volume, the glucose saccharification yield after a simple irradiation was relatively lower than that obtained by chemical methods. Therefore, more research on irradiation pretreatment in combination with other pretreatment methods should be conducted to make this option economically viable.

In this report, we demonstrated the effectiveness of a combined pretreatment method using gamma ray irradiation (GRI) and low concentration of aqueous alkali solution (1 % NaOH) in enhancing the enzymatic digestibility of RS. Hydrolysis yield and element composition were analyzed after the pretreatment. Enzymatic hydrolysis of the pretreated RS was optimized. Finally, the rice straw hydrolysate (RSH) was used as a carbon source for *Chlorella protothecoides* cultivation under heterotrophic and mixotrophic conditions to examine the effects of the RSH on cell growth, biomass and lipid concentrations, and the cellular fatty acid profile was analyzed. Microalga has been considered as one of the potential sources for sustainable and biodegradable biodiesel production due to their high oil content, strong adaptive capacity to environment, shorter life cycles than energy crops that are used in biodiesel production, and no occupation for cropping area [14]. As far as we are concerned, this is the first study to investigate the cultivation of the oleaginous microalga *C. protothecoides* using RSH for lipid production.

## Results and discussion

### Compositional change in pretreated RS

To enhance the glucose recovery from RS, the GRI and alkali (1 % NaOH) pretreatment methods were applied. Weight loss and composition change in biomass are important indices to evaluate the effectiveness of pretreatment methods. Therefore, changes in composition and weight loss in the raw and pretreated RS biomass were first analyzed to compare the effects of single and combined pretreatment methods.

As seen in Table 1, when compared with the composition of the untreated RS, alkali pretreatment alone could increase the cellulose content by 40.5 %, while the xylan, lignin, extractives, and water contents were reduced by 42.9, 23.4, 10.8, and 44.3 %, respectively. But, the combined pretreatment with GRI and alkali showed further increase in the cellulose content up to 60 % depending on the dose of irradiation. In contrast, the levels of other components were decreased depending on the radiation dose. The combined pretreatment increased the removal percentage of xylan, lignin, extractives, and water up to 63.8, 40.2, 32.3 and 58.7 %, respectively. There were no detectable sugar degradation products such as furfural and hydroxymethylfurfural, which are known to inhibit cell growth [4]. Lignin limits enzyme access to carbohydrates by imposing a physical barrier and by causing unproductive binding of enzymes, and hemicellulose is thought to restrict the access of enzyme to cellulose in pretreated biomass [15]. Therefore, the increase of cellulose content and reduction of xylan and lignin can promote the process of enzymatic hydrolysis. Since a large amount of lignin and xylan content was removed after the combined pretreatment, the results clearly indicated that the combined pretreatment could enhance the enzymatic digestibility of RS.

**Table 1:** Effect of pretreatments of rice straw on its main components

Pretreatment conditions	Composition analysis of rice straw (w/w, %)^a^				
	Cellulose	Xylan	Klason lignin	Water-soluble extractives	Water content
Untreated	37.5	10.5	18.4	9.3	9.7
25 kGy	37.1	10.8	18.2	9.2	9.6
50 kGy	37.4	10.6	18.3	9.5	9.3
75 kGy	37.3	10.3	18.2	9.4	9.1
100 kGy	37.9	10.6	18.5	9.4	9.1
NaOH	52.7	6.0	14.1	8.3	5.4
25 kGy + NaOH	52.7	5.9	13.6	8.1	5.5
50 kGy + NaOH	54.2	5.8	12.5	8.1	4.3
75 kGy + NaOH	56.9	4.4	11.5	7.2	4.3
100 kGy + NaOH	60.0	3.8	11.0	6.3	4.0

^a^Results are expressed as an average percentage of the components obtained from triplicate assays

In addition, a significant change in the concentration of minerals in neutralized RS after the combined pretreatment was identified by ICP methods (Table 2). Among the minerals, potassium, calcium and magnesium were the three major elements in the untreated RS. However, the potassium content was greatly reduced (22-fold) after the combined pretreatment while calcium, magnesium, and manganese contents were increased. The sodium content was also increased after the pretreatment which might be due to the alkali pretreatment. Trace amounts of non-essential heavy metals, which can cause an inhibitory effect on the cell growth during fermentation, such as arsenic, cadmium and lead were also identified [16]. The results indicated that extracted elemental composition could be changed by pretreatment and the analysis may give the insight for eventual problems during the fermentation process.

**Table 2:** Elemental composition before and after the combined pretreatment

Elements (mg/Kg)	Untreated	100 kGy + NaOH
Al	156.83 ± 8.12	58.29 ± 5.65
As	1.21 ± 0.03	ND^a^
Ca	1605.93 ± 21.30	2805.35 ± 123.53
Cd	0.06 ± 0.01	0.07 ± 0.01
Cr	30.44 ± 1.43	14.30 ± 0.36
Cu	2.71 ± 0.06	6.46 ± 0.41
Fe	185.21 ± 6.36	215.27 ± 15.74
K	3743.71 ± 0.32	169.11 ± 12.96
Mg	629.97 ± 7.06	1016.06 ± 46.19
Mn	304.73 ± 12.51	515.47 ± 48.62
Mo	0.33 ± 0.01	ND^a^
Na	98.70 ± 0.95	383.19 ± 17.76
Ni	2.84 ± 0.19	2.01 ± 0.1
Phosphate	356.86 ± 5.83	73.55 ± 4.95
Pb	3.22 ± 0.02	5.12 ± 1.48
Sulfate	304.12 ± 1.09	277.20 ± 12.93
Zn	38.06 ± 0.43	55.49 ± 3.51

Data are expressed as means of triplicate measurements ± standard deviations

^a^Below the detection limit

### Effect of pretreatment on enzymatic hydrolysis

**Fig. 1:**
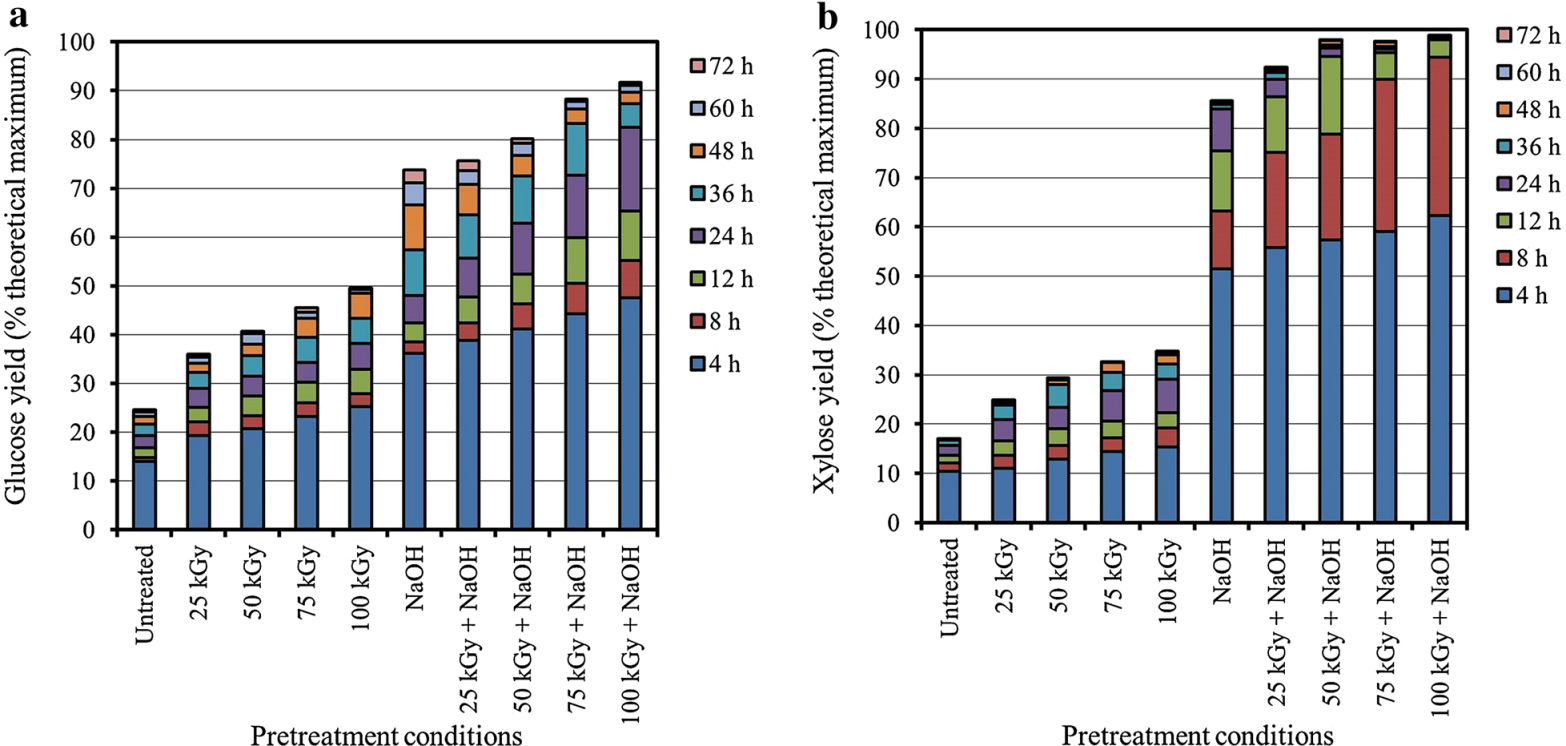
Effect of gamma irradiation on the pretreatment of rice straw with NaOH. **a** glucose saccharification yield based on the theoretical maximum and **b** xylose saccharification yield

In general, the efficiency of enzymatic hydrolysis of biomass increases when combination of enzymes such as cellulase, xylanase and glucosidase are used rather than cellulase alone [5]. In this study, enzymatic hydrolysis was carried out with the enzymes, Cellic CTec2 (6 filter paper unit [FPU]/g RS) and Cellic HTec2 (12 fungal xylanase unit [FXU]/g RS). Cellic HTec2 was used to break down the hemicellulose barrier for efficient enzymatic hydrolysis of cellulose in RS. The theoretical glucose and xylose saccharification yields (based on the weight of RS after pretreatment) after enzymatic hydrolysis are illustrated in Fig. 1. The initial hydrolysis rate (at 4 h) in all of the RS samples was much higher than the subsequent hydrolysis rate, which was consistent with other studies; and selective initial hydrolysis of amorphous cellulose or subsequent insufficiency of new catalytic sites might be the possible explanation for this phenomenon [17, 18]. The glucose and xylose saccharification yields of the GRI-pretreated RS increased in a radiation-dose-dependent manner, and glucose was the main component of the enzymatic RSHs. Higher glucose and xylose saccharification yields up to twofold were achieved by GRI pretreatment than those in the untreated RS. However, conversion of cellulose and xylan into their monomeric forms was still less than 50 %, and a similar result was also presented in previous reports [8]. Although this method is very simple, the results indicated that the GRI pretreatment alone was insufficient for pretreatment of RS. Alkali pretreatment resulted in higher glucose and xylose saccharification yields than GRI pretreatment and the conversion yield of cellulose and xylan into their monomeric forms after 72 h was 74.2 and 85.8 %, respectively. To improve the conversion yield, alkali pretreatment was carried out after GRI. The result clearly showed that the combined pretreatment was a highly effective method for pretreatment of RS. The maximum conversion yields of cellulose and xylan after 72 h were 92.3 and 98.9 %, respectively. To the best of our knowledge, these values represent the highest conversion yield of RS into monomeric sugars [12, 18–20]. Although the enzymes used in this study, from Novozymes, cannot be used for a large-scale production of lipid, this result can still be useful for the application of saccharification process with commercial enzymes.

### Effect of enzyme loading

**Fig. 2:**
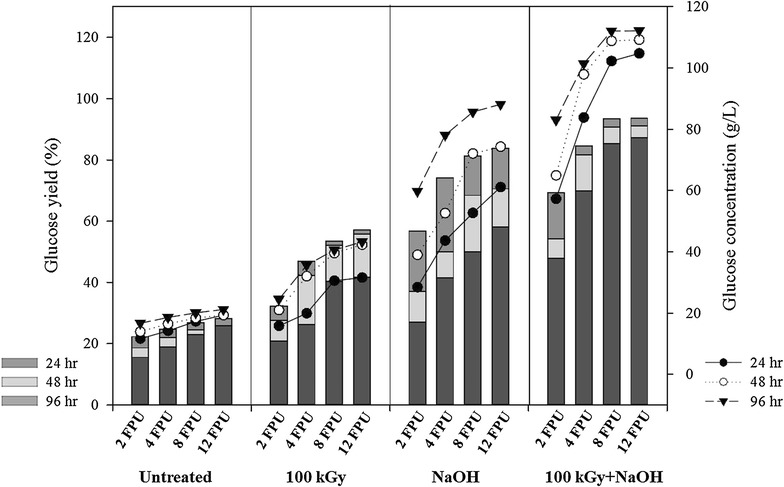
Effect of Cellic CTec2 loading on the hydrolysis of pretreated rice straw and glucose concentration in hydrolysate

In many reports, addition of accessory enzymes such as β-glucosidases and hemicellulose has been performed to increase the hydrolysis yield of lignocellulose [19]. In this report, the effect of Cellic CTec2 loading on the hydrolysis of RS, especially in conversion of cellulose to glucose, was investigated and the result is shown in Fig. 2. Higher Cellic CTec2 loading increased the initial conversion of cellulose. The effect of enzyme concentration on cellulose conversion was not significant in the untreated RS. Conversion of cellulose in the untreated RS was 22 % with the use of 2 FPU, and it was increased to 28 % with 12 FPU. The effect of enzyme concentration was more significant in the RS pretreated with alkali than that pretreated with GRI. When the gamma-irradiated RS was digested, the conversion yield was increased from 32 % with the use of 2 FPU of the enzyme to 56 % with 12 FPU. In the case of the alkali-pretreated RS, the conversion yield was found to increase from 56 % with the use of 2 FPU of the enzyme to 84 % with 12 FPU.

When 2 FPU of the enzyme was added to the hydrolysis reaction of the RS pretreated with GRI and alkali, the conversion of cellulose was only 47 % after 24 h of digestion. At 8 FPU, a theoretical glucose saccharification yield of 85.2 % could be achieved after 24 h. Further hydrolysis reaction caused an increase of only 5.4 and 2.7 % in the theoretical glucose saccharification yield after 48 and 96 h, respectively. Although the concentration of the enzyme was further increased to 12 FPU, the increase in glucose saccharification yield was only 2 % more compared to that with 8 FPU at 24 h.

### Growth of *C. protothecoides* in RSHs medium

**Fig. 3:**
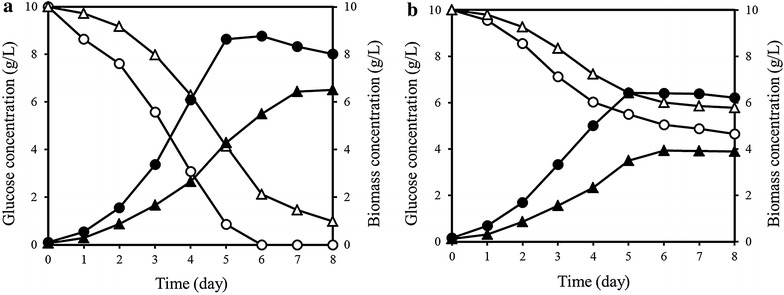
Growth and lipid content of *C.* *protothecoides* from glucose and rice straw hydrolysate. *Open* and *closed circles* the residual glucose concentration and cell concentration in glucose, respectively. *Open* and *closed triangles* the residual glucose concentration and cell concentration in the rice straw hydrolysate, respectively. **a** heterotrophic culture condition with an equivalent glucose concentration of 10 g/L and **b** mixotrophic culture condition with an equivalent glucose concentration of 10 g/L

To investigate the possible utilization of RS for the production of lipid, the RSH prepared from the RS pretreated with the combination of alkali and GRI (100 kGy) was used as a carbon source in the medium for the growth of *C. protothecoides* (Fig. 3a). When the RSH was used as a sole carbon source (heterotrophic culture, equivalent to 10 g/L glucose), the cell concentration reached 6.5 g/L after cultivation for 8 days. When 10 g/L of purified glucose was added to the medium, the maximum cell concentration was about 8.7 g/L. The difference of final cell concentration between RSH and purified glucose could be caused by the impurities in the RSH. Growth of *C. protothecoides* in the RSH medium was also carried out under the mixotrophic conditions (Fig. 3b). In this mixotrophic condition, glucose source was also added to the medium under light. The final cell concentration was 4 g/L in the mixotrophic culture, which was lower than the final cell concentration of 6.5 g/L in the heterotrophic culture. However, the substrate conversion to cell mass was about 92 % in the mixotrophic culture, which was higher than 65 % in the heterotrophic culture. The cell concentration was also higher with the use of purified glucose than with the use of RSH in the mixotrophic culture. The lower microalgal growth in the mixotrophic culture was demonstrated in the lipid production by *Chlorella vulgaris* [21]. Under mixotrophic growth conditions, addition of 5 and 10 % glucose exerted inhibitory effect on the growth. This phenomenon was also observed for *C. protothecoides* on feeding with glucose in the range between 1.5 and 6 % [22]. However, the effects of glucose on microalgal growth have not been intensively explored.

Because RS was one of the abundant food wastes especially in Northeast Asia, this result could suggest that RS could be utilized to replace the expensive carbon source for the cultivation of biomass. However, the cost of preparation and energy balance of process are also important. Therefore, further work on economical valuation of whole process including pretreatment, saccharification, fermentation and purification should be carried out. It has been reported that the cost contribution of hydrolysis enzyme to ethanol production was found to be $1.47/gal if the yield was based on the previous report. When the saccharification was at maximum theoretical yield, the cost contribution decreased to $0.68/gal [23]. Therefore, the increased yield of the hydrolysis with irradiation could reduce the production cost of biofuel from RS.

### Comparison of algal lipid production between glucose and RSH media

The total lipid content and fatty acid composition were compared between the cultures grown in glucose and RSH as carbon sources. Fatty acid composition by fatty acid methyl ester (FAME) analysis is shown in Table 3. In heterotrophic culture, the total lipid content and fatty acid composition were not significantly different between *C. protothecoides* grown in glucose medium and in RSH medium. Total lipid content was about 55 % and total fatty acid content was 45 % based on the cellular dry weight. 9-Octadecenoic acid was the most abundant fatty acid in *C. protothecoides*, and hexadecanoic acid, 9,12-octadecadienoic acid (Z,Z), and octadecatrienoic acid were the main fatty acids. In the mixotrophic culture, there was no difference in the total lipid content and fatty acid composition between *C. protothecoides* grown in glucose medium and in RSH medium. The total lipid content was the same between *C. protothecoides* in heterotrophic culture and in mixotrophic culture, but there was a marginal decrease in the total fatty acid content of *C. protothecoides* in the mixotrophic culture, which was not statistically significant. With respect to the fatty acid composition, the 9-octadecenoic acid content was decreased, but the 9,12,15-octadecatrienoic acid content was increased. It was reported that the lipid content of *C. vulgaris* was also the same between the heterotrophic and mixotrophic cultures [21]. Therefore, this result shows that RSH can be utilized as a carbon source to replace glucose without causing any changes in the fatty acid content and composition. Microbial oils are produced by some oleaginous microorganisms, such as yeast, fungi, bacteria, and microalgae. Many yeast species, such as *Cryptococcus albidus*, *Lipomyces starkeyi*, and *Saccharomyces cerevisiae*, were found to be able to accumulate oils under some cultivation conditions. The lipid-producing yeast has the advantages of fast growing and easy gene manipulation. However, microalgae are drawing much attention because of the utilization of carbon dioxide [14].

**Table 3:** Fatty acid composition of lipid extracts of *C. protothecoides* in different carbon sources

Components	Relative FAME content (%)^a^			
	Heterotrophic cultivation		Mixotrophic cultivation	
	Glucose	RSH	Glucose	RSH
7,10-Hexadecadienoic acid, methyl ester	2.13	2.30	9.02	1.64
7,10,13-Hexadecatrienoic acid, methyl ester	3.11	3.91	4.17	5.90
Hexadecanoic acid, methyl ester	15.38	16.26	20.40	20.56
Heptadecanoic acid, methyl ester	0.47	0.60	0.59	0.48
9,12-Octadecadienoic acid (Z,Z)-, methyl ester	16.91	17.10	10.52	11.59
9-Octadecenoic acid, methyl ester	51.37	47.57	31.81	34.77
9,12,15-Octadecatrienoic acid, methyl ester	9.57	10.93	21.76	23.14
Octadecanoic acid, methyl ester	1.05	1.32	1.74	1.92
Total lipid/cell dry weight (%)	55.77	54.97	56.37	55.10
Total fatty acid/cell dry weight (%)	45.62	45.37	40.95	40.04
Total fatty acid/total lipid (%)	81.80	82.54	72.65	72.66
Total fatty acid yield (g/L)	4.00	2.95	2.63	1.57

^a^FAME composition is reported as weight percent of all FAMEs

## Conclusions

In this study, rice straw was investigated for the production of lipid from microalgae. For the efficient hydrolysis of rice straw, combined gamma irradiation and NaOH pretreatment methods were employed. Gamma irradiation further increased the conversion yield of glucose induced by NaOH pretreatment. Although slight growth retardation was observed in RSH medium, the lipid content and fatty acid composition of *C. protothecoides* were the same as in glucose-based medium. This result suggests that rice straw can be utilized as an alternative carbon source for the production of microalgal lipid, and the pretreatment with irradiation can enhance the efficacy of hydrolysis of lignocellulosic biomass.

## Methods

### Microalga and raw material

The microalgal strain used in this study was the *C. protothecoides* strain 25 from the UTEX algae culture collection (Texas University of Austin, TX, USA). Bold’s basal medium (BBM) containing yeast extract (3 g/L) was used as the basic medium for cultivation [24].

Organically grown RS was collected from the fields near Jeongeup, Republic of Korea. Before pretreatment, the RS was chopped (~5 cm), washed thoroughly under tap water to remove the extraneous matter, oven-dried at 70 °C until constant weight was achieved, and then packed in a polystyrene bag for further use.

### Pretreatments

The chopped RS was filled into thin plastic envelopes and exposed to the desired absorbed dose of gamma rays at a dose rate of 10 kGy/h in a ^60^cobalt facility of Korea Atomic Energy Research Institute (Jeongeup, Republic of Korea). For alkaline pretreatment, 10 g of RS was mixed with 90 mL of 1.0 % (w/v) NaOH solution and the mixture was incubated for 5 days at room temperature (25–30 °C). After pretreatment, the wet RS was collected by filtration and washed with deionized water until neutral pH. All the pretreated RS samples were oven-dried at 70 °C until a constant weight was achieved. Prior to performing subsequent procedures, all of the RS samples were ground to pass through a 420-um sieve using a pulverizer (Korea medi, Daegu, Republic of Korea).

### Composition analysis

The compositions of the oven-dried natural and pretreated RS were analyzed by following a two-stage acid hydrolysis protocol developed by the National Renewable Energy Laboratory. Briefly, each sample was subjected to hydrolysis with 72 % sulfuric acid at 30 °C for 1 h and then hydrolysis with 3 % sulfuric acid at 121 °C for 1 h. The autoclaved hydrolysis solution was neutralized to a pH of 6.0 by adding calcium carbonate and was vacuum filtered. The sugars released by acid hydrolysis and enzymatic hydrolysis were quantified on a high-performance liquid chromatography (HPLC) system (Agilent 1200, Agilent Technologies, Santa Clara, CA, USA) equipped with a refractive index detector (Agilent 1260, Agilent Technologies) and a Bio-Rad Aminex HPX-87P column (Bio-Rad Laboratories, Hercules, CA, USA) was used with HPLC grade water at a flow rate of 0.6 mL/min at 65 °C. All of the samples were filtered through a 0.20-μm filter and diluted with an eluent before analysis on HPLC. Various concentrations of the pure monomeric sugar were used for standards. Theoretical glucose and xylose contents were calculated from glucose and xylose contents multiplied by conversion factors of 0.90 and 0.88, respectively, to account for the mass gained during the hydrolysis of glucan and xylan. The moisture content was measured as the weight loss of 1 g RS samples dried at 105 °C to a constant weight. Acid insoluble lignin (Klason lignin) content was defined as the weight of ash-free, oven-dried filter cake at 105 °C to a constant weight. Residue recovery, delignification, and loss of cellulose and xylan in GRI and/or alkali-treated RS samples were determined gravimetrically using the untreated RS samples as the control. The elemental composition was determined with an inductively coupled plasma (ICP) method by Korea Basic Science Institute (Daejeon, Republic of Korea).

### Enzymatic hydrolysis

Enzymatic hydrolysis of the RS samples was carried out using Cellic CTec2 and Cellic HTec2 (Novozymes, Bagsvaerd, Denmark). The filter paper unit (FPU) of Cellic CTec2 (120 FPU/mL) was pre-determined by manufacturer. The fungal xylanase unit (FXU) of Cellic HTec2 (2500 FXU/mL) provided by the manufacturer was used to calculate the loading. Enzymatic hydrolysis was performed in 50 mM sodium citrate buffer (pH 4.8) containing meropenem (Sigma-Aldrich, St. Louis, CA, USA) at a concentration of 12.5 mg/L. Substrate consistency was maintained at 20 % (w/v). The RS samples were soaked in the buffer and heated for 30 min prior to the addition of enzymes. The reactions were initiated by mixing the indicated amount of enzymes followed by incubation at 50 °C in a shaking (200 rpm) water bath (Jeio Tech, Daejeon, Republic of Korea). The progression of enzymatic hydrolysis was measured at regular intervals by estimating reducing sugars in the RSH using HPLC following the above-described protocol. All the experiments were performed in triplicates and the average values were represented.

### Batch fermentations

Batch cultivation was carried out in a 500-mL baffled flask containing 150 mL of BBM supplemented with 10 g of a carbon source (pure glucose or the same amount of glucose by adding appropriate volumes of the RSH), and 3 g of yeast extract per liter was used as a nitrogen source instead of NaNO_3_. The pH of the medium was adjusted to 6.8, and the alga (~5 % V/V) was inoculated and cultivated in an orbital shaker with shaking (120 rpm) at 25 °C. A 12/12-h on/off light cycle (200 µmol m^−2^ s^−1^) was applied for the mixotrophic culture, while heterotrophic cultivation was maintained in the dark.

### Measurement of biomass and total lipid content

The microalgal growth was determined by measuring the optical density of the culture broth using a UV–Vis spectrophotometer at 540 nm (Biochrome, Cambridge, UK) using the corresponding blank media as the control. Algal biomass was calculated by the following regression equation that was empirically obtained: *y* = 0.5398*x* − 0.1604 (*R*^2^ = 0.9998, *p* < 0.05), where *y* (g/L) is the dry cell weight, *x* is the absorbance of the suspension at 540 nm. For dry weight measurement of microalgal biomass, the microalgal culture was filtrated through GF/C glass fiber filter (GE Healthcare, Little Chalfont, UK) and then washed to remove medium components. The filter containing the biomass was dried in a forced convection oven (Jeio Tech) at 70 °C to a constant weight.

After cultivation, the algal cells were harvested by centrifugation (4000 rpm) for 15 min, and the cell pellets were freeze-dried in a high vacuum for 2 days. Then, 0.2 g of the dried algae was mixed with 0.5 mL of distilled water and 3 mL of chloroform/methanol (2:1, v/v), and the mixture was shaken for 20 min, centrifuged (10,000 rpm) for 10 min and the chloroform phase was collected in a pre-weighed tube. The samples were extracted five times and dried in a dry block bath (MG-2000, Eyela, Tokyo, Japan) by purging with nitrogen gas at 60 °C. Lipid content was expressed as a percentage of lipid weight per biomass weight.

### Fatty acids analysis

Fatty acids were extracted from 50 mg dried algal cells with 1 mL of NaOH–CH_3_OH. To each aliquot, 0.1 mL of an internal standard of 10 g/L tridecanoic acid (C13:0) dissolved in chloroform was added for fatty acid quantification. The mixture was shaken for 10 min at 75 °C in a water bath, and cooled to room temperature. Then, 2 mL of boron trifluoride–methanol solution (1:2, v/v) was added, and the mixture was shaken for 10 min at 75 °C and cooled to room temperature. Later, 0.3 mL of saturated salt solution was added to make a layered solution. Subsequently, 2 mL of hexane was added, and the mixture was centrifuged. The upper fatty acids layer was subjected to GC-linked mass spectrometry analysis on an Agilent HP 6890N machine equipped with a 5975 inert mass selective detector. A capillary column DB-5MS (Length 30 m, I.D. 0.25 mm, Thickness 0.25 µm, Agilent Technologies) was used. The sample size was 1 µL and the carrier gas was helium at a flow rate of 1.0 mL/min, the solvent delay time was 5 min and the injection temperature was 250 °C. The initial oven temperature was 130 °C for 1 min, and it was increased to 200 °C at a rate of 5 °C/min and this temperature was maintained for 5 min. Fatty acids were identified by a direct comparison of their mass spectral pattern and retention index with the NIST 05 mass spectral database. Data are reported as an average of triplicate measurements.

## Abbreviations

FAME: fatty acid methyl ester
FPU: filter paper unit
FXU: fungal xylanase unit
GC: gas chromatography
GRI: gamma ray irradiation
HPLC: high-performance liquid chromatography
RS: rice straw
RSH: rice straw hydrolysate
